# Comparative Studies of Salivary and Blood Sialic Acid, Lipid Peroxidation and Antioxidative Status in Oral Squamous Cell Carcinoma (OSCC)

**DOI:** 10.12669/pjms.303.4985

**Published:** 2014

**Authors:** Mahmood Rasool, Saima Rubab Khan, Arif Malik, Khalid Mahmood Khan, Sara Zahid, Abdul Manan, Mahmood Husain Qazi, Muhammad Imran Naseer

**Affiliations:** 1 Mahmood Rasool, Center of Excellence in Genomic Medicine Research (CEGMR),King Abdulaziz University, Jeddah, Saudi Arabia.; 2 Saima Rubab Khan, Institute of molecular biology and biotechnology,The University of Lahore, Lahore, Pakistan.; 3 Arif Malik, Institute of molecular biology and biotechnology,The University of Lahore, Lahore, Pakistan.; 4 Khalid Mahmood Khan, Fatima Jinaah Medical College, Sir Ganga Ram Hospital, Lahore, Pakistan.; 5 Sara Zahid, Institute of molecular biology and biotechnology,The University of Lahore, Lahore, Pakistan.; 6 Abdul Manan, Institute of molecular biology and biotechnology,The University of Lahore, Lahore, Pakistan.; 7 Mahmood Husain Qazi, Centre for Research in Molecular Medicine, The University of Lahore, Lahore, Pakistan.; 8 Muhammad Imran Naseer, Center of Excellence in Genomic Medicine Research (CEGMR),King Abdulaziz University, Jeddah, Saudi Arabia.

**Keywords:** Neoplastic, Squamous Cell Carcinoma, Lipid peroxidation, MDA

## Abstract

***Objective*** ***:*** Oral squamous cell carcinoma (OSCC) is considered to be a serious life threatening issue for almost two decades. The objective of this study was to evaluate the over production of lipid peroxidation (LPO) byproducts and disturbances in antioxidant defense system in the pathogenesis of oral cancer.

***Methods*** ***:*** Lipid peroxidation and antioxidant status in OSCC patients were estimated and compared the sensitivity and specificity of circulating biomarkers (MDA, Sialic acid, Catalase, SOD, GSH and Neuraminidase) with β-2 microglobulin (β-2MG) at different thresholds in blood and saliva using receiver operating characteristics (ROC) curve design.

***R*** ***esults*** ***:*** Our results showed that the levels of MDA and Sialic acid were significantly increased in plasma of OSCC patients as compared to healthy subjects whereas antioxidant level was significantly decreased.

***Conclusion*** ***: ***ROC analysis indicated that MDA in saliva is a better diagnostic tool as compared to MDA in blood and β-2MG in blood is better diagnostic marker as compared to β-2MG level in saliva.

## INTRODUCTION

Oral squamous cell carcinoma (OSCC) is among the most frequently seen of all oral cancers.^1^ Oral cancer is twofold more prevalent in men than in women mainly affecting adult males due to high percentage of alcohol consumption and tobacco habits ranging from sixty to seventy years of age.^2^ Globally, 90% of oral cancers are OSCC which are differentiated by typical neoplastic cells located all through the epithelium and outside the basement membrane.^2^^-^^4^

OSCC is categorized as a malignant tumor that invades the jaw bone. Histologically, the normal epithelial cells are easily perceptible while abnormal cells show variability in nuclear size and shape, increased nuclear-cytoplasmic ratio, increased amount of keratin and abnormal mitotic activity. The only treatment of choice is the surgical removal but it compromises the quality of life as it disturbs both the normal function and esthetics of the patient. Intraorally, OSCC develops frequently in the tongue (20-30%), floor of the mouth (15-20%), retromolar and tonsilar pillar areas 15%, soft palate (10-15%), buccal mucosal (10%), alveolar bone (10%) and maxillary sinus (15-20%).^2^^,^^5^^-^^8^ Multiple factors affect the etiology of OSCC in which genetic and environmental factors play potent roles. Epidemiological studies indicate the multiplicative effect of tobacco consumption and alcohol containing more than 300 carcinogenic chemicals including polycyclic aromatic hydrocarbons with respect to frequency and duration on OSCC.^9^ Microsomal complex enzymes which are chemically hydrocarbon hydroxylases convert these polycyclic aromatic hydrocarbons into carcinogens which are responsible for tumor suppressor genes and DNA repairing genes which ultimately develop OSCC.^10^ Considerable association of premalignant and malignant oral lesion and peritonsillar cancers (Base of the tongue and palative tonsils) with other viruses such as herpes simplex virus (HSV) and Epstein Barr Viruses (EBV) has been reported by.^11^^,^^12^ A case control study of 201 patients supported the detection of HPV DNA in 19% of the total cases and 5% of controls and 43% of peritonsillar cancers.^13^ Free radical scavengers such as antioxidants (vitamin A, C and β carotene act as chemopreventive agents against oral cancer. At molecular level, transforming growth factor alpha (TGF-α) is expressed by carcinoma cells and this simultaneous expression of TGF-α and Epidermal Growth Factor Receptor (EGFR) by the carcinoma cells result in the proliferation of cells forming abnormal cancerous cells.^14^ Gene therapy that target specific genes concerned in the upregulation of cancer could be considered for cancer treatment in the near future.

## METHODS

***Place of Work: ***All the experiment work was done at the institute of Molecular Biology and Biotechnology, The University of Lahore. Prior to the start of study, informed consent was taken from all the participants. The study was approved from the local Ethical Committee of the University.

***Experiment Design: ***Individuals were divided into four groups. Group 1 included the blood of health individuals n=10, Group 2 was OSCC patients (blood of diagnosed OSCC patients) n=30, Group 3 was control for saliva of health individuals n=10, Group 4 was OSCC patients (Saliva of diagnosed OSCC) patients n=30.

***Exclusion Criteria: ***In this study we excluded the patients with associated illness like Myocardial Infarction, Hypertension, Renal, Hepatic, Pancreatic and Pulmonary diseases were excluded from the study.

***Sample Collection: ***Total of 5.0ml of venous blood was drawn from healthy controls and histopathologically diagnosed OSCC patients. Similarly unstimulated whole saliva samples were taken between 9:00am to 11:00am from both healthy controls and histopathologically diagnosed OSCC patients. 

***Blood Analysis: ***Blood was centrifuged at 4000 rpm for 10 minutes and serum was separated. Blood samples were collected into EDTA tubes.

***Biochemical Analysis of Samples: ***The sample were processed and analyzed for the estimation of Glutathione (GSH), Catalases, Super Oxide Dismutase (SOD), Malondialdehyde (MDA), Sialic acid and Neuraminidase by the spectrophotometric method.

***β2-Microglobulin Assay: ***β2-Microglobulin assay was done by commercially available Signosis β2-MG ELISA kit.

## RESULTS

The MDA levels were obtained 3.15±0.58 (µmol/ml) and 4.55±1.483 (µmol/ml) in control and patient groups respectively from blood samples which were significant p<0.05, also in saliva samples the MDA levels were 0.19±0.02 (µmol/ml) and 0.5433±0.258 (µmol/ml) in control and patient groups respectively (Table Ia, Ib).

**Fig.1:**
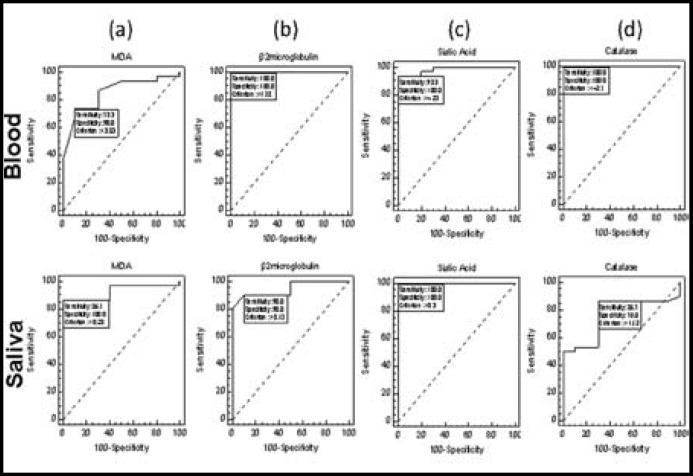
Sensitivity and specificity of MDA (a) Beta_2_-microgobulin (b) Sialic acid (c) and Catalase (d) in blood and saliva of the control and patients of OSCC

For checking the sensitivity and specificity of MDA levels at different thresholds ROC analysis was done separately in both blood and saliva (Fig.1a). The optimum threshold values of MDA level in blood and saliva obtained were >3.53 and >0.23 respectively. Whereas sensitivity of MDA obtained was high 86.67% (69.3% - 96.2%) in saliva and low 73.33% (54.1% - 87.7%) in blood. The specificity record of MDA levels obtained were 100% (69.2% -100%) in saliva and 90% (55.5% - 99.7%) in blood. Other than sensitivity and specificity the AUC computed in saliva was 0.927 (0.798 - 0.985) statistically significant (P <0.01) and in blood the AUC was 0.852 (0.704 -0.944) statistically significant (P <0.01). It clarified that MDA in saliva is better diagnostic test as compared to MDA in blood. 

The Beta_2_-microgobulin levels obtained were 1.39 ± 0.22 (µg/ml) and 2.74±0.83 (µg/ml) in control and patient groups respectively from blood samples which were significant p<0.05, also in saliva samples the Beta_2_-microgobulin levels were 0.79 ± 0.05 (µg/ml) and 0.71 ± 0.40 (µg/ml) in control and patient groups respectively (Table Ia, Ib).

For checking the sensitivity and specificity of Beta_2_-microgobulin levels at different thresholds ROC analysis was done separately in both blood and saliva (Fig.1b). The optimum threshold values of Beta_2_-microgobulin levels in blood and saliva were >1.78 and >0.13 respectively. Whereas sensitivity of Beta_2_-microgobulin levels obtained were low i.e. 90% (73.5% - 97.9%) in saliva and high i.e. 100% (88.4% - 100.0%) in blood. The specificity record of Beta_2_-microgobulin levels obtained were 90% (55.5% - 99.7%) in saliva and 100% (69.2% - 100%) in blood. Other than sensitivity and specificity the AUC computed in saliva was 0.945 (0.824 - 0.992) statistically significant (P <0.01) and in blood the AUC was 1.00 (0.912 -1.00) statistically significant (P <0.01), which states that Beta_2_-microgobulin in blood is better diagnostic test as compared to Beta_2_-microgobulin level in saliva.

The sialic acid levels obtained were 2.58 ±0.80 (µg/L) and 5.52±1.371 (µg/L) in control and patient groups respectively from blood samples which were significant p<0.05, also in saliva samples the sialic acid levels were 0.16 ± 0.08 (µg/L) and 1.88 ± 0.73 (µg/L) in control and patient groups respectively (Table Ia, Ib).

ROC analysis was done for sensitivity and specificity of sialic acid separately in both blood and saliva (Fig.1c). The optimum threshold values of sialic acid level in blood and saliva were > 4.23 and >0.30 respectively. Whereas sensitivity of sialic acid levels were high i.e. 100% (88.4% - 100%) in saliva and low i.e. 93.33% (77.9% - 99.2%) in blood. The specificity record of sialic acid levels were 100% (69.2% -100%) in both saliva and blood. Other than sensitivity and specificity the AUC computed in saliva was 1.00 (0.912 - 1.00) statistically significant (P <0.01) and in blood the AUC was 0.983 (0.882 - 1.00) statistically significant (P <0.01). It showed that sialic acid levels in both, saliva and blood were equally important diagnostic tests.

Further, Catalase levels were obtained 4.29±0.83 (µmol/mol of protein) and 0.75±0.63 (µmol/mol of protein) in control and patient groups respectively from blood samples which were significant p<0.05. In saliva samples the catalase levels were 1.14±0.16 (µmol/mol of protein) and 1.17±0.70 (µmol/mol of protein) in control and patient groups respectively (Table Ia, Ib).

ROC analysis for catalase was done separately in both blood and saliva (Fig.1d). The optimum threshold values of catalase levels in blood and saliva were <= 2.7 and >1.12 respectively. Where sensitivity of catalase levels obtained were low 86.67% (69.3% - 96.2%) in saliva and high 100% (88.4% - 100%) in blood. The specificity record of catalase levels obtained were 70% (34.8% - 93.3%) in saliva and 100% (69.2% - 100%) in blood. Other than sensitivity and specificity the AUC computed in saliva was 0.765 (0.604 - 0.884) statistically significant (P <0.01) and in blood the AUC was 1.00 (0.912 - 1.00) statistically significant (P <0.01). It proves that catalase in blood was better diagnostic test as compared to catalase in saliva and catalase levels of blood showed equal sensitivity and specificity in comparison to Beta_2_-microgobulin in blood.

The Superoxide Dismutase (SOD) levels obtained were 0.92±1.79 (ng/ml) and 0.15±0.10 (ng/ml) in control and patient groups respectively from blood samples which were significant p<0.05, also in saliva samples these levels were 01.16± 0.25 (ng/ml) and 0.613 ± 0.251 (ng/ml) in control and patient groups respectively (Table Ia, Ib).

**Fig.2:**
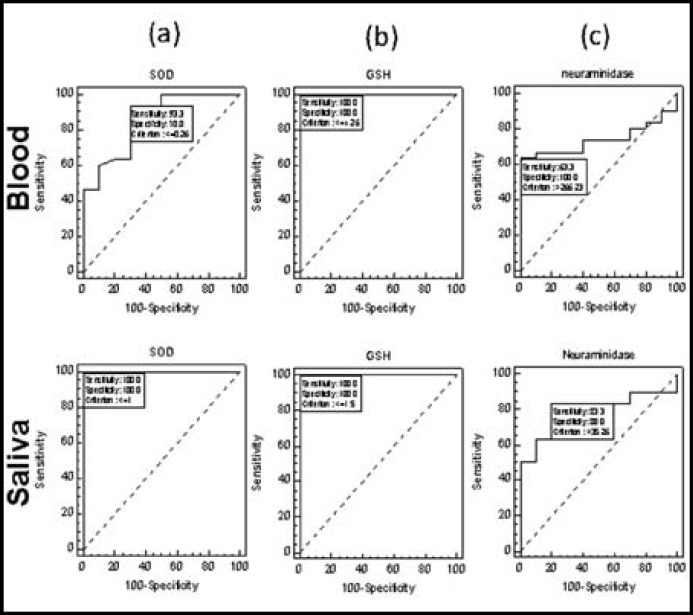
Sensitivity and specificity of Superoxide dismutase (SOD) (a) GSH (b) and Neuraminidase (c) in blood and saliva of the control and patients of OSCC

ROC analysis for SOD was done separately in both blood and saliva (Fig.2a). The optimum threshold values of SOD levels in blood and saliva were <=0.26 and <=1 respectively. Whereas sensitivity of SOD level obtained were high 100% (88.4% - 100%) in saliva and low 93.3% (77.9% - 99.2%) in blood. The specificity record of SOD levels obtained were 100% (69.2% -100%) in saliva and 70% (34.8% - 93.3%) in blood. Other than sensitivity and specificity the AUC computed in saliva was 1.00 (0.912 - 1.00) statistically significant (P <0.01) and in blood the AUC was 0.858 (0.712 -0.948) statistically significant (P <0.01). It meant that SOD in saliva was better diagnostic test as compared to SOD in blood.

The GSH levels obtained were 9.82±1.32 (mg/dl) and 2.40±0.77 (mg/dl) in control and patient groups respectively from blood samples which were significant p<0.01. In saliva samples the GSH levels were 2.090±0.245 (mg/dl) and 0.884 ±0.257 (mg/dl) in control and patient groups respectively (Table Ia, Ib).

For checking the sensitivity and specificity of GSH levels at different thresholds ROC analysis was done separately in both blood and saliva (Fig.2b). The optimum threshold values of GSH levels in blood and saliva were <=4.26 and <=1.5 respectively. Whereas same levels of GSH sensitivity were obtained i.e. 100% (88.4%-100.0%) in saliva and blood. The specificity record of GSH levels were also same i.e. 100% (69.2% -100%) in saliva and blood. Other than sensitivity and specificity the AUC computed in saliva and blood was 1.00 (0.912 - 1.00) statistically significant (P <0.01). It meant that GSH levels in saliva and blood were equally reliable diagnostic tests.

The neuraminidase levels obtained were 233.15±36.13 (mg/100ml) and 279.18±73.75 (mg/100ml) in control and patient groups respectively from blood samples which were significant (p<0.05). In saliva samples the neuraminidase levels were 32.86±9.46 (mg/100ml) and 48.36±15.31 (mg/100ml) in control and patient groups respectively (Table Ia, Ib).

ROC analysis for neuraminidase was done as shown in (Fig. 2c). The optimum threshold values of neuraminidase levels in blood and saliva were >266.23 and >35.26 respectively. Whereas, sensitivity of neuraminidase level was high 83.3% (65.3% - 94.4%) in saliva and low 63.33% (43.9% - 80.1%) in blood. The specificity record of neuraminidase levels obtained were 80% (44.4% -97.5%) in saliva and 100% (69.2% - 100%) in blood (Table-IIa). Other than sensitivity and specificity the AUC computed in saliva was 0.800 (0.644 - 0.909) statistically significant (P <0.01) and in blood AUC was 0.737 (0.574 -0.863) statistically significant (P <0.01, Table-IIb). These results confirm that neuraminidase is much sensitive in saliva but more specific in blood. But we cannot compare neuraminidase with Beta_2_-microgobulin.

## DISCUSSION

Cancer is fundamentally an occasion start from gene level and finally leads to the DNA damage. Numerous factors play important role in carcinogenesis such as chemicals, viruses, irradiation and genetic composition of an individual. Whereas, ROS and RNS are two important factors which leads to DNA damage. The extent of DNA damage depends not only on ROS/RNS levels but also on the body’s resistance mechanisms alongside a variety of cellular antioxidants.

Lipid peroxidation depends upon the level of Lipid Hydroperoxides (LHP) and MDA. In this study, our experimental results in oral cancer patients showed increased levels of MDA which may attributed to increased configuration or insufficient clearance of free radicals by the cellular antioxidants. Previously, it was hypothised that increased levels of lipid peroxidation was the result of large amount of free radicals produce by the cancer cells^15^ and show a strong relationship with free radical activity and malignancy.^16^

**Table-Ia:** T-test for MDA, SOD, GSH, Catalase, β-2 Microglobulin, Neuraminidase and Sialic Acid in saliva of OSCC patients.

	*Group*	*N*	*Mean±SD*	*P value*
MDA (µmol/ml)	Control saliva	10	0.19±0.02	.000
	OSCC saliva	30	0.54±0.25	
SOD (ng/ml)	Control saliva	10	1.16±0.10	.000
	OSCC saliva	30	0.61±0.25	
GSH (mg/dl)	Control saliva	10	2.09±0.24	.000
	OSCC saliva	30	0.88±0.25	
CAT (µmol/mol of protein)	Control saliva	10	1.14±0.16	.000
	OSCC saliva	30	1.74±0.70	
β2-microglobulin (µg/ml)	Control saliva	10	0.08±0.05	.000
	OSCC saliva	30	0.71±0.40	
Neuraminidase (mg/100ml)	Control saliva	10	32.86±9.46	.001
	OSCC saliva	30	48.36±15.31	
Sialic Acid (µg/L)	Control saliva	10	0.16±0.08	.000
	OSCC saliva	30	1.88±0.73	

**Table-Ib:** T-test for MDA, SOD, GSH, Catalase, β-2 Microglobulin, Neuraminidase and Sialic Acid in blood of OSCC patients

	*Group*	*N*	*Mean±SD*	*P value*
MDA (µmol/ml)	Control blood	10	3.15±0.58	.000
	OSCC blood	30	4.55±1.48	
SOD (ng/ml)	Control blood	10	0.92±1.79	.210
	OSCC blood	30	0.15±0.10	
GSH (mg/dl)	Control blood	10	9.82±1.32	.000
	OSCC blood	30	2.40±0.77	
CAT (µmol/mol of protein)	Control blood	10	4.29±0.83	.000
	OSCC blood	30	0.75±0.63	
β2-microglobulin (µg/ml)	Control blood	10	1.39±0.22	.000
	OSCC blood	30	2.74±0.83	
Neuraminidase (mg/100ml)	Control blood	10	233.15±36.13	.014
	OSCC blood	30	279.18±73.75	
Sialic Acid (µg/L)	Control blood	10	2.58±0.80	.000
	OSCC blood	30	5.52±1.37	

**Table-IIa:** Comparison of Sensitivity and specificity of circulating biochemical markers (MDA, sialic acid, catalase, SOD, GSH and neuraminidase) with β-2 microglobulin (standard) in OSCC

		*Sensitivity*	*Specificity*	*AUC*
Beta_2_-microgobulin	Saliva	90%	90%	0.945
	Blood	100%	100%	1.00
MDA	Saliva	87.6%	100%	0.927
	Blood	73.3%	90%	0.852
Sialic Acid	Saliva	100%	100%	1.00
	Blood	93.3%	100%	0.983
Catalase	Saliva	86.7%	70%	0.765
	Blood	100%	100%	1.00
SOD	Saliva	100%	100%	1.00
	Blood	93.3%	70%	0.858
GSH	Saliva	100%	100%	1.00
	Blood	100%	100%	1.00
Neuraminidase	Saliva	83.3%	80%	0.800
	Blood	63.3%	100%	0.737

**Table-IIb:** AUC and P value of Circulating Biomarkers in Blood and Saliva

		AUC	95% CI^a^	P (Area=0.5)
MDA	Blood	0.852	0.704-0.944	<0.0001
	Saliva	0.927	0.798-0.985	<0.0001
β2-MG	Blood	1.000	0.912-1.000	0.000
	Saliva	0.945	0.824-0.992	<0.0001
Sialic Acid	Blood	0.983	0.882-1.000	<0.0001
	Saliva	1.000	0.912-1.000	0.000
Neuraminidase	Blood	0.737	0.574-0.863	0.0020
	Saliva	0.800	0.644-0.909	<0.0001
SOD	Blood	0.858	0.712-0.948	<0.0001
	Saliva	1.000	0.912-1.000	0.000
Catalase	Blood	1.000	0.912-1.000	0.000
	Saliva	0.765	0.604-0.884	0.001
GSH	Blood	1.000	0.912-1.000	0.000
	Saliva	1.000	0.912-1.000	0.000

a = Binomial exact, AUC = Area under the ROC curve,

 Furthermore, non-protein thiol such as GSH in conjugation with glutathione-S-transferase (GST) and glutathione peroxidase (GPx), plays a important role in defensive mechanism of cells against ROS.^17^ In our study a major reduction of plasma GSH observed reflects enhanced pro-oxidant level of the cells and interact with the increased lipid peroxides in the patients with oral cancer. The damaging toxic effects of free radicals is prevented by antioxidative enzymes such as SOD, CAT and GPx play important role inside the cell by directly reacting with oxygen free radicals. GPx is a selenium dependent antioxidative enzyme which carry out the degradation of both H2O_2_ and LHP by using GSH due to which intracellular DNA damage is inhibited responsible for carcinogenesis.^18^ Previously, oxidative damage to the cell membrane has been reported to inactivate GPx.^19^

 Both increase^20^ and decrease^21^^-^^23^ in CAT activity have been reported previously. In this study decrease in CAT activity was observed which may be due to increased nitric oxide (NO) end products, endogenous production of the superoxide anion, or decreased activity of GPx and SOD or may be due to the all of these factors. Moreover, it might also be due to a higher amount of oxidative stress, because all patients involved in this study were in advanced clinical stages (stage III/IV) with tumor. Vitamin C along with vitamin E prevents the oxidation of GSH which is required for regeneration of both vitamin C and vitamin E, and GSH in oral cancer patients might be responsible for the low levels of these antioxidants. Evidence of the role of NO• in carcinogenesis showed that both constitutive nitric oxide synthase (cNOS) and inducible nitric oxide synthase (iNOS) are detected in various human cancers.^24^^,^^25^ Previously, it is also reported that biopsy samples in a high-grade tumor of human breast cancer showed the presence of increased expression of iNOS.^26^

 Previously, oxidative stress and lipid peroxidation have been reported as main causes of inflammation and tissue damage.^23^ Further, it is concluded that oxidative stress is increased due to increased level of lipid peroxidation and nitric oxide products which compromised antioxidant defense in patients with oral cancer.

 In our study the significantly increased levels of serum MDA and decreased levels of serum Total antioxidant status (TAS) in oral malignancy patients showed high statistical significance as compared with healthy individuals and directly reflects increased oxidative stress and lipid peroxidation. Thus measurement of MDA in serum and total antioxidant status, an extent of lipid peroxidation and antioxidant level, may be helpful in understanding the severity of the disease in oral malignancies.

## CONCLUSION

Our results showed that in saliva GSH is at the top of list as a diagnostic biomarker of OSCC. It is followed by SOD, sialic acid and β-2 microglobulin. Salivary sialic acid and oxidative stress could serve as sensitive markers of OSCC. Finally, Saliva is equally reliable biomarker as blood is considered and GSH, Sialic acid and SOD may be used as a cost effective diagnostic biomarker for OSCC.
